# Network Intrusion Detection Method Based on FCWGAN and BiLSTM

**DOI:** 10.1155/2022/6591140

**Published:** 2022-04-13

**Authors:** Zexuan Ma, Jin Li, Yafei Song, Xuan Wu, Chen Chen

**Affiliations:** ^1^College of Air and Missile Defense, Air Force Engineering University, Xi'an 710051, China; ^2^Xi'an Satellite Control Center, Xi'an 710043, China

## Abstract

Imbalanced datasets greatly affect the analysis capability of intrusion detection models, biasing their classification results toward normal behavior and leading to high false-positive and false-negative rates. To alleviate the impact of class imbalance on the detection accuracy of network intrusion detection models and improve their effectiveness, this paper proposes a method based on a feature selection-conditional Wasserstein generative adversarial network (FCWGAN) and bidirectional long short-term memory network (BiLSTM). The method uses the XGBoost algorithm with Spearman's correlation coefficient to select the data features, filters out useless and redundant features, and simplifies the data structure. A conditional WGAN (CWGAN) is used to generate a small number of samples in the dataset, add them to the original training set to supplement the dataset samples, and apply BiLSTM to complete the training of the model and realize the classification. In comparative tests based on the NSL-KDD and UNSW-NB15 datasets, the accuracy of the proposed model reached 99.57% and 85.59%, respectively, which is 1.44% and 2.98% higher than that of the same type of CWGAN and deep neural network (CWGAN-DNN) model, respectively.

## 1. Introduction

The continuous development of computer and network technology has greatly improved people's lives, but with it come a variety of attacks and threats at the network level, making network security an unavoidable and urgent problem. As an effective method to detect and defend against network attacks, the intrusion detection system (IDS) has been widely used. It monitors network traffic in real time, classifies it as normal or malicious, and provides information necessary to intrusion prevention systems. In recent years, machine learning and deep learning have been widely used for intrusion detection. However, since real-life network traffic data are unbalanced and relatively little has malicious attack attributes, the training sets of such methods are severely unbalanced. Hence, while existing network intrusion detection systems have high resolution accuracy for whether there is an attack, the detection accuracy of various samples is still low, especially for minority-class attacks, resulting in the misclassification of such traffic as other traffic, and the failure to meet performance analysis requirements. Therefore, it is important to solve the network data imbalance problem and improve the performance of model intrusion detection.

The class imbalance problem is commonly solved by enhancing the model training effect by increasing the number of samples in datasets, and much research has been conducted based on this method. Maryam Yousefnezhad et al. [1] proposed a feature extraction ensemble classification method based on deep learning. Firstly, the feature selection algorithm based on ensemble margin is used to select the samples, and the deep learning method is used to extract the sample features. Finally, the outputs of multiple KNN and SVM are combined according to Dempster–Shafer method. This method uses the method of ensemble learning, which can improve the detection rate of attack types to a great extent. At the same time, feature selection based on ensemble margin can remove the useless data in the original dataset, and improve the overall detection accuracy, and shorten the training time to a certain extent. However, the structure is complex, there are many classifiers, and the overall calculation cost is high. Meanwhile, this method uses KNN and SVM as classifiers to classify samples, and the overall classification accuracy of the model has a large space for improvement. Considering the complexity of dimensions and the low efficiency of traditional algorithms, a chaotic cuckoo optimization algorithm with levy flight, disruption operator, and opposition-based learning (CCOALFDO) is proposed by Kelidari and Hamidzadeh [2]. The algorithm combines levy flight, disruption operator and opposition-based learning to select the optimal feature subspace for classification. Levy flight can deal with uncertainty and better update the cuckoo steps in high-dimensional space. The opposition-based learning and disruption operator can improve the search ability of the algorithm and ensure the diversity of the population. The algorithm proposed in this paper combines the above advantages, which can greatly reduce the randomness of feature selection and avoid falling into the local optimal solution. At the same time, due to the elimination of some redundant features, the classification accuracy can be greatly improved. However, the combination of multiple algorithms leads to the increase of the overall computational complexity of the algorithm, which requires higher computational cost, slows down the convergence speed and increases the computational time. Gonzalez-Cuautle et al. [3] proposed a resampling method that integrates the synthetic minority oversampling technique (SMOTE) and grid search algorithms to solve the problems of overfitting and low classification accuracy. This method improved the classification results of the intrusion detection system (IDS) dataset by merging synthetically generated balanced data and adjusting different supervised learning algorithms. SMOTE can oversample the data sample and increase the number of minority data. The grid search algorithm can automatically optimize the parameters, and find the parameters with the best detection effect and apply them to the model structure, and avoid falling into the local optimal solution, which ensures the optimality of the model detection effect. However, SMOTE randomly synthesizes the original data according to the *k*-nearest neighbor principle, does not learn the essence of the original data, and the quality of the generated samples is poor. At the same time, the grid search algorithm searches every parameter, which leads to too large calculation cost, too long calculation time, and there is a large space for improvement. Lee and Park [4] proposed AE-CGAN-RF, a model to solve the data imbalance problem by using an autoencoder to reduce the dimension of the network traffic and a conditional generative adversarial network (CGAN) to generate data samples, which were passed to a random forest (RF) to complete the intrusion detection classification. The model could greatly improve the accuracy of minority class sample detection, and reduces the data dimension, which reduces the time required for training and reduces the calculation cost. However, the use of RF as a classifier led to a low overall detection accuracy because of RF's weak classification ability. Lee and Park [5] proposed a detection model using a generative adversarial network (GAN) to generate minority class attack samples and RF for classification. This method increases the minority samples of CIC-IDS2017 dataset and improves the detection ability of the model for minority attack samples, so that the model can achieve better detection effect. At the same time, the structure is simple and the detection speed is fast. However, only ordinary GAN is used for sample generation, without considering the instability of the GAN, there are hidden dangers in the process of sample generation, and other datasets and models were not used to further validate its feasibility, which is not convincing. Liu et al. [6] proposed a GAN-FS method to address feature redundancy. The model can select dataset features based on feature variance, eliminate the impact of redundant data and useless data on the model detection effect to a great extent, improve the accuracy and speed of detection, and uses a GAN to generate samples, which increase the number of samples and enhance the training effect. Comparative experiments confirmed that the method could effectively improve model detection performance, but the method does not consider the degrees of freedom of GAN training, and the generated data are unsupervised and uncontrollable. Compared with CGAN, it is less targeted. At the same time, it only selects the features according to the feature variance, and the detection method is not comprehensive, which has certain limitations. He [7] addressed the low accuracy of class imbalance data detection and proposed a model using a conditional Wasserstein generative adversarial network (CWGAN) to generate minority class attack samples and a Deep Neural Networks (DNN) as a classifier for network intrusion detection, which improves the detection effect compared to a DNN. However, only using DNN as classifier to identify intrusion behavior, there is still a large gap in detection accuracy compared with other deep learning methods. At the same time, the high dimensionality of data is not considered, and the use of the network intrusion detection system in a large-scale network environment will be limited by time and space complexity because the data have high dimensionality and nonlinear characteristics. Therefore, dimensionality reduction for high-dimensional data is a key step to improve detection speed and performance.

To solve the above problems, this paper combines feature selection with a CWGAN. The feature selection-based dimensionality reduction of high-dimensional data can filter out redundant and useless features, simplify the data structure, improve intrusion detection performance, and decrease training time. The CWGAN oversamples the minority class data to supplement the samples and balance the data distribution, thus improving detection performance. A bidirectional long short-term memory network (BiLSTM) is used to extract and classify the features from the time series. The loss function and optimization algorithm are analyzed to select the most suitable hyperparameters.

This paper makes the following contributions:

1. We propose FCWGAN-BiLSTM, a network intrusion detection system based on FCWGAN and a BiLSTM network, to alleviate the impact of class imbalance on detection performance and improve the overall performance of a network intrusion detection model
2. We use XGBoost and Spearman correlation coefficients for feature selection to filter out redundant and useless data and simplify the feature structure, which reduces computational difficulty and improves detection accuracy
3. We apply CWGAN to generate minority class samples to supplement the dataset, enhance the model training effect, reduce the impact of class imbalance on the detection rate, and improve detection performance
4. A BiLSTM network captures information in network traffic data with long-term dependency, extracts network traffic feature extraction based on time series, and effectively uses future moment information to improve the model classification effect
5. Model performance analysis experiments, model ablation experiments, and comparison experiments with different data augmentation algorithms and classification algorithms demonstrate the performance of the proposed model

The rest of this paper is organized as follows.  Section 2 presents the background and related work.  Section 3 presents the proposed model, Section 4 provides experimental results and analysis, and Section 5 presents the conclusions.

## 2. Background and Related Work

### 2.1. Feature Selection

Feature selection is a method of selecting relevant features of a dataset by obtaining a subset from the original feature set based on specific criteria. Data dimensionality reduction is often applied to high-dimensional complex data [8]. Unlike feature extraction, feature selection preserves the physical meaning of the original features by retaining some of the data, and thus makes the model more readable and interpretable [9, 10]. In the field of intrusion detection, where datasets are characterized by a large volume of data and high dimensionality, feature selection reduces computational difficulty and eliminates data redundancy [11], thereby improving the detection rate of the model and reducing false positives. For example, a firefly algorithm was used for feature selection and to pass the generated features through a classifier based on C4.5 and a Bayesian network (BN) to complete the classification for intrusion detection [12]. The method selected important features in the KDD CUP 99 dataset and reduced the 41-dimensional features to 10 dimensions, which achieved better detection performance and reduced computation. However, the method suffers from a low discovery rate and slow solution speed, which leads to long calculation times. Le et al. [13] proposed SFSDT, a feature selection model that combines a hybrid sequence forward selection (SFS) algorithm with a decision tree (DT) model to select the best feature subset from the complete set of features in a dataset. The CF function in the SFS algorithm is adjusted, and the accuracy and error score of the DT model on each feature subset are generated by the SFS. SFSDT starts from an empty set and sequentially adds features to enhance the accuracy of the DT model until it is maximized on a validation dataset (feature subset). The algorithm reduces execution time and required memory, and significantly improves detection performance. However, SFS can only add features, and cannot remove them, and it tends to fall into local optima. Thus, it requires a large number of experiments to obtain the best subset. Considering the above problems, we use XGBoost and the Spearman correlation coefficient for dataset feature selection.

#### 2.1.1. XGBoost

Proposed by Chen in 2015, XGBoost (eXtreme Gradient Boosting) is a model framework based on the idea of the gradient boosting decision tree (GBDT) [14]. It has the advantages of high speed, high efficiency, and strong performance, and has been widely used to solve classification and regression problems. The core idea is to generate a new tree by splitting the features in a dataset, and then to add new trees. It fits the residual of its last prediction to obtain a new function and improves performance through iteration. The traditional GBDT algorithm uses only first-order derivative information, while XGBoost uses a second-order Taylor expansion of the loss function and a regular term to speed up training and prevent overfitting. We use this method to rank the importance of features in the dataset [15].

#### 2.1.2. Spearman Correlation Coefficient

We use the Spearman correlation coefficient to measure the correlation between features. Proposed by Spearman in 1904, it measures the strength of the relationship between two variables [16], and it takes values in the range (−1, 1). The Spearman correlation coefficient between variables *x*_*i*_ and *y*_*i*_ is calculated as

(1) $$\begin{array}{c} \rho =\frac{\sum_{i}\left(x_{i}-\bar{x}\right)\left(y_{i}-\bar{y}\right)}{\sqrt{\sum_{i}{\left(x_{i}-\bar{x}\right)}^{2}\sum_{i}{\left(y_{i}-\bar{y}\right)}^{2}}}, \end{array}$$

where *x*_*i*_(*i*=1,2,…, *n*) and *y*_*i*_(*i*=1,2,…, *n*) are elements of the vectors *X* and *Y*, respectively. A value of *ρ* close to ±1 indicates a strong association; hence one of the features can be filtered out. A value close to 0 indicates that there is no association between them, and both should be retained.

### 2.2. CWGAN

A GAN is a deep learning model inspired by the two-person zero-sum game in game theory and is used to simulate complex high-dimensional distributions of real-world data. It consists of a generator (*G*) and discriminator (*D*) [17], which are both neural networks. The generator captures the potential distribution of real data samples and generates new data samples. The discriminator is a binary classifier used to determine whether the input sample is real or generated data. The classification results are passed back to the generator and discriminator through updates of the weighted loss. The above networks are trained until the discriminator can no longer distinguish between real and generated samples [18]. Its optimization process is a minimax game problem with the goal to achieve a Nash equilibrium so that the generated network can estimate the distribution of the data samples [19]. The objective function for generating the adversarial network is

(2) $$\begin{array}{c} \underset{G}{\text{min}}\underset{D}{\text{max}}V\left(D,G\right)=E_{x∼p_{\text{data}}\left(x\right)}\left[\log D\left(x\right)\right]+E_{z∼p_{\text{data}}\left(z\right)}\left[\log\left(1-D\left(G\left(z\right)\right)\right)\right], \end{array}$$

where *p*_data_ denotes the distribution of real samples, the function *G*(*z*) maps noise *z* to the data space, and *D*(*x*) is the probability that sample *x* is real data. To distinguish between real and generated data, *D*(*x*) should be as large as possible, and *D*(*G*(*z*)) as small as possible.

The CGAN is based on a GAN, where category information and noise are merged with the original data as the input to the generator and discriminator [20], with loss function

(3) $$\begin{array}{c} \underset{G}{\text{min}}\underset{D}{\text{max}}V\left(D,G\right)=E_{x∼p_{\text{data}}\left(x\right)}\left[\log D\left(x|y\right)\right]+E_{z∼p_{\text{data}}\left(z\right)}\left[\log\left(1-D\left(G\left(z|y\right)\right)\right)\right], \end{array}$$

where *y* represents the category information, and other parameters are the same as in (2).

A GAN is different from ordinary oversampling, as it generates new samples by obtaining the potential distribution of the original data and passing it randomly into the generator. By training the generator and discriminator, the generated samples are similar to the original sample distribution with high confidence. GANs are used to generate samples for minority classes and to expand datasets. For example, the SIGMA method [21] generates new samples to enhance the ability of IDSs to resist new types of attacks, combining a GAN with hybrid local search and genetic algorithms to iteratively generate new samples to retrain the intrusion detection system based on machine learning until the detection rate converges. AEGAN [22] is a hybrid model consisting of adversarial environment reinforcement learning (AE-RL) and a CGAN, whose model is trained on a network intrusion detection dataset to generate synthetic samples to deal with class imbalance problems. The above methods can improve the performance of network intrusion detection systems, but none considers the vanishing gradient problem that might occur during the training of GANs.

GANs and CGANs can generate samples and reduce class imbalance problems. However, their use of Jensen-Shannon scatter requires overlap between the distributions of real and generated samples, which is nonexistent or negligible when the discriminator is trained to be optimal, which can lead to model collapse and vanishing gradient problems [23].

**Figure 1:**
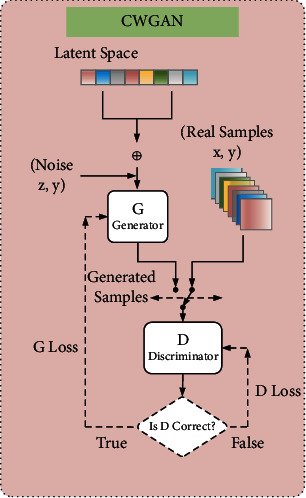
CWGAN workflow diagram.

To solve the above problems, we introduce the Lipschitz limit and Wasserstein distance to CGAN to realize CWGAN for the dataset samples, with the workflow shown in Figure 1.

We fix the discriminator, input the noise vector and labels to the generator, and train it to simulate the real data distribution. We use the discriminator to judge the real and generated samples. If it cannot distinguish between them, we fix the generator and train the discriminator, and if it can, we fix the discriminator and train the generator. We repeat these steps until the loss function of the discriminator is stabilized at about 0.5, at which time we generate attack samples and add them to the training set.

Through the above method, the model can generate data of a specified pattern to supplement the dataset, while effectively avoiding the vanishing gradients caused by the failure of the discriminator to converge during training. The objective function of CWGAN is

(4) $$\begin{array}{c} V\left(D,G\right)=\underset{D}{\text{max}}\left\{E_{x∼p\text{data}}\left[D\left(x|y\right)\right]-E_{x∼p_{g}}\left[D\left(x|y\right)\right]-\lambda E_{x∼p_{\text{Penaty}}}{\left[\left\|\nabla_{x}D\left(x|y\right)\right\|-1\right]}^{2}\right\}, \end{array}$$

where *λ* is an artificial parameter, ‖∇_*x*_*D*(*x*)‖ is the calculation paradigm for *x* in *D*(*x*), and *x* ~ *p*_Penaty_ is the middle position of the line connecting points on *p*_*r*_ and *p*_*g*_.

### 2.3. BiLSTM

**Figure 2:**
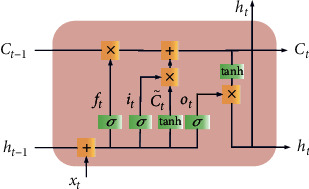
LSTM structure diagram.

The model in a traditional neural network focuses only on the processing of the current moment, while a recurrent neural network (RNN) can use information processed at the current moment at the next moment [24]. Considering the problem of the vanishing gradient and gradient explosion during the training of an RNN, Hochreiter et al. proposed the long short-term memory network (LSTM) [25], which adds a gate mechanism and a memory unit on the basis of the RNN and memory unit to effectively solve the problems of RNNs, and better solves the longer distance dependence problem [26]. LSTM has input, forget, and output gates, as shown in Figure 2.

The LSTM structure is described as

(5) $$\begin{array}{c} \left\{\begin{array}{c} f_{t}=\;\sigma \left(W_{f}\cdot \left[h_{t-1},x_{t}\right]+b_{f}\right)\\ i_{t}=\;\sigma \left(W_{i}\cdot \left[h_{t-1},x_{t}\right]+b_{i}\right)\\ {\tilde{C}}_{t}=\text{ tan}h\left(W_{C}\cdot \left[h_{t-1},x_{t}\right]+b_{C}\right)\\ C_{t}=\;f_{t}\cdot C_{t-1}+i_{t}\cdot {\tilde{C}}_{t}\\ o_{t}=\;\sigma \left(W_{o}\cdot \left[h_{t-1},x_{t}\right]+b_{o}\right)\\ h_{t}=\;o_{t}\cdot \text{tan}h\left(C_{t}\right) \end{array}\right., \end{array}$$

where *f*_*t*_ is the forget gate; *i*_*t*_ is the input gate; ${\tilde{C}}_{t}$ and *C*_*t*_ are the current input and unit state, respectively; *σ* is the sigmoid function; *W*_*f*_, *W*_*i*_, *W*_*o*_, and *W*_*C*_ are the weight matrices of the forget gate, input gate, output gate, and current input unit state, respectively; [*h*_*t*−1_, *x*_*t*_] denotes the concatenation of the two vectors; and *b*_*f*_, *b*_*i*_, *b*_*o*_, and *b*_*C*_ are the bias terms of the forget gate, input gate, output gate, and current input unit state, respectively. The above parameters change continuously during training.

Considering the distinct temporal characteristics of network traffic data, the use of RNN-like approaches to deal with network intrusion problems has unique advantages. For example, in [27], a deep learning-based intrusion detection system, DL-IDS, uses a hybrid network of convolutional neural networks (CNNs) and LSTM to extract the spatiotemporal characteristics of network traffic data, thus providing a better intrusion detection system. However, it was not considered that the unidirectional LSTM can only read sequence data from one direction and cannot exclude the influence of subsequent information on the detection results. Thus, BiLSTM was used instead of LSTM to process incoming data [28].

**Figure 3:**
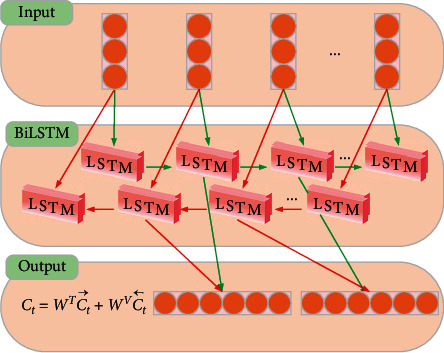
BiLSTM process diagram.

BiLSTM combines forward and backward LSTM to learn from forward and backward time-series data. The hidden layer contains two units with the same input that are connected to the same output, where one processes the forward time series, and the other the backward time series, increasing the time series involved in training by learning features better, thus providing higher accuracy for longer time series. The BiLSTM process is shown in Figure 3.

The process is

(6) $$\begin{array}{c} \left\{\begin{array}{c} {\overset{⟶}{C}}_{t}=\text{LSTM}\left(x_{t},{\overset{⟶}{h}}_{t-1},{\overset{⟶}{C}}_{t-1}\right)\\ {\overset{\leftarrow }{C}}_{t}=\text{LSTM}\left(x_{t},{\overset{\leftarrow }{h}}_{t-1},{\overset{\leftarrow }{C}}_{t-1}\right)\\ C_{t}=W^{T}{\overset{⟶}{C}}_{t}+W^{V}{\overset{\leftarrow }{C}}_{t} \end{array}\right., \end{array}$$

where the LSTM function represents the nonlinear transformation of the input feature, which is encoded as the corresponding hidden state of the LSTM ((5) and *W*^*T*^ and *W*^*V*^ are the weight coefficients corresponding to the forward and backward moment unit state, respectively.

## 3. Network Intrusion Detection Method Based on FCWGAN and BiLSTM

**Figure 4:**
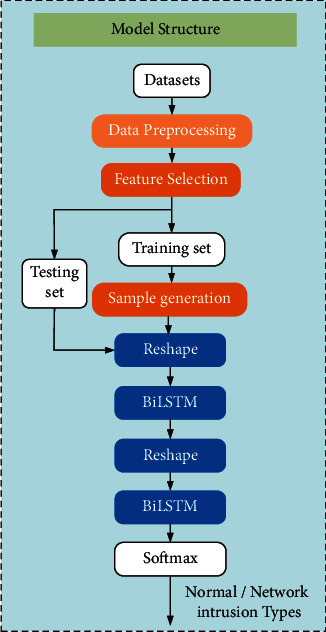
Schematic diagram of model structure based on FCWGAN and BiLSTM.

We propose a network intrusion detection method based on FCWGAN and BiLSTM. XGBoost is used in the feature selection stage to rank the importance of the features in the dataset, whose relevance is analyzed based on the Spearman correlation coefficient. Features with strong relevance and low importance are filtered out to simplify the feature structure. The selected features are passed into CWGAN together with the labels, and minority class samples in the training set are generated in a controlled manner. Generated samples are passed into BiLSTM together with the original data in the training set for training, and the model is validated on a test set. The intrusion detection process includes stages of data preprocessing, feature selection, sample generation, feature extraction and training, and testing, as shown in Figure 4.

### 3.1. Data Preprocessing

Tag encoding was used to convert the string-type features in the NSL-KDD and UNSW-NB15 datasets to numeric-type. It was judged whether there was a null value in the dataset, and if there was none, the data were normalized by Min − Max,

(7) $$\begin{array}{c} x=\frac{x-M_{\text{min}}}{M_{\text{max}}-M_{\text{min}}}, \end{array}$$

where *M*_min_ and *M*_max_ are the minimum and maximum values, respectively, of the dimension.

### 3.2. Feature Selection

In the feature selection stage, we used XGBoost to rank the feature importance, and Spearman's correlation coefficient to analyze the feature relevance. Irrelevant and redundant features were filtered out, and important features were retained to improve detection speed and enhance detection results.

XGBoost obtains a new function by fitting the residuals of the last prediction of the model and iterates to improve model performance [29]. Unlike the traditional GBDT algorithm that uses only first-order derivative information, the XGBoost algorithm performs a second-order Taylor expansion on the loss function and adds a regularization term to improve the model training speed and prevent overfitting. The target loss function of the XGBoost algorithm is

(8) $$\begin{array}{c} \left\{\begin{array}{c} \text{Obj}=\sum_{i=1}^{n}l\left(y_{i},{\hat{y}}_{i}\right)+\sum_{k=1}^{K}\Omega \left(f_{k}\right)\\ \Omega \left(f_{k}\right)=\gamma T+\frac{1}{2}\lambda {\left\|\omega \right\|}^{2} \end{array}\right., \end{array}$$

where $l\left(y_{i},{\hat{y}}_{i}\right)$ is the loss function, which represents the difference between the predicted value ${\hat{y}}_{i}$ and true value *y*_*i*_; and Ω(*f*_*k*_) aims to prevent overfitting, where *T* is the number of child nodes, *ω* denotes the leaf weights, *γT* reduces the number of leaf nodes in the tree, *γ* is the penalty coefficient, *λ*‖*ω*‖^2^ is the regularization term, and *λ* is the regularization coefficient.

XGBoost requires several iterations to continuously generate the tree [30], assuming that the t-th iteration produces the tree, and the objective function of the t-th iteration is

(9) $$\begin{array}{c} {\text{Obj}}^{\left(t\right)}=\sum_{i=1}^{n}l\left(y_{i},{\hat{y}}_{i}^{\left(t-1\right)}+f_{t}\left(x_{i}\right)\right)+\Omega \left(f_{t}\right). \end{array}$$

where Ω(*f*_*t*_) is a function to prevent overfitting.

We can evaluate the reasonableness of the decision tree structure based on the structure loss,

(10) $$\begin{array}{c} {\text{Obj}}^{\left(t\right)}\left(p\right)=-\frac{1}{2}\sum_{j=1}^{T}\frac{\left(\sum_{i\in I_{j}}g_{i}\right)}{\sum_{i\in I_{j}}h_{i}+\lambda }+\gamma T, \end{array}$$

where *g*_*i*_ and *h*_*i*_ are the first- and second-order derivatives of the loss function to the predicted values after iteration t-1, *I*_*j*_={*i|p*(*x*_*i*_)=*j*} is the index of leaf node *j*, and a smaller structural loss indicates a better decision tree structure.

If the tree splits at node *j*, the structure gain of the leaf node is

(11) $$\begin{array}{c} {\text{Obj}}_{s}=\text{Obj}\left(p_{\text{before}}\right)-\text{Obj}\left(p_{\text{after}}\right)=\frac{1}{2}\left(\frac{{\left(\underset{i\in I_{j}}{\sum }g_{i}\right)}^{2}}{\underset{i\in I_{j}}{\sum }h_{i}+\lambda }+\frac{{\left(\underset{i\in I_{L}}{\sum }g_{i}\right)}^{2}}{\underset{i\in I_{L}}{\sum }h_{i}+\lambda }+\frac{{\left(\underset{i\in I_{R}}{\sum }g_{i}\right)}^{2}}{\underset{i\in I_{R}}{\sum }h_{i}+\lambda }\right)-\gamma , \end{array}$$

where *γ* is the split coefficient, which can reduce the complexity of the model and prevent overfitting. This split gain is used to judge the quality of the split node.

Based on the above formulas, the importance of the features was ranked, and their relevance was analyzed through the Spearman correlation coefficient.

The importance of the features is sorted according to formula (11), and the Spearman correlation coefficient is used to analyze the feature correlation. The two are combined to eliminate irrelevant and redundant features, filter out key features, and pass them to the GAN for minority class sample generation.

### 3.3. Sample Generation

In the sample generation process, CWGAN was trained using noise and data samples that underwent feature selection and preprocessing [31], as shown in Table 1.

In the process of training CWGAN, the generator and discriminator were trained in turn, as follows:

1. The discriminator is fixed and the generator is trained to simulate the distribution of the real data
2. The generator is fixed, and the discriminator is trained until it cannot distinguish whether samples are from the real dataset or the generator
3. The discriminator is fixed, and the generator is trained until the discriminator cannot distinguish samples by successive training
4. Steps 1–3 are repeated until the loss value of the discriminator reaches 0.5
5. The generator is used to generate attack samples, and these are added to the training set to complete sample generation

### 3.4. Feature Extraction and Training

In the feature extraction stage, a BiLSTM layer learned the long-term temporal features in the dataset, Nadam optimization was applied to the neural network [32], a dropout layer alleviated overfitting, and a softmax classifier was used for network attack classification.

### 3.5. Testing

The trained model was used to classify the test set to obtain the prediction type. To ensure credible test results, the model was tested by *k*-fold cross-validation. The softmax function,

(12) $$\begin{array}{c} \sigma {\left(x\right)}_{j}=\frac{e^{x_{j}}}{\sum_{k=1}^{K}e^{x_{k}}}\;j=1,\ldots ,K, \end{array}$$

was used to calculate the probability of the classification and compare it with the original labels.

## 4. Experiment and Result Analysis

### 4.1. Experimental Settings

The performance of network intrusion detection methods based on FCWGAN and BiLSTM were evaluated according to the following experiments:

  Experiment 1: model performance analysis
  Experiment 2: model noise robustness
  Experiment 3: model ablation
  Experiment 4: comparison of data enhancement algorithms
  Experiment 5: comparison of classification algorithms
  Experiment 6: comparison of intrusion detection models

The experimental environment was a 64-bit Windows 10 operating system with TensorFlow learning framework, an AMD Ryzen 9 5900HX with Radeon Graphics at 3.30 GHz, and 32 GB RAM.

A Bayesian optimization algorithm was used for automatic optimization of model parameters, whose settings are shown in Table 2.

The categorical cross-entropy loss function is

(13) $$\begin{array}{c} \text{oss}=\;-\frac{1}{N}\sum_{i=0}^{N}\left(y_{i}\log y_{i}+\left(1-y_{i}\right)\log\left(1-y_{i}\right)\right). \end{array}$$

### 4.2. Dataset and Experimental Evaluation Criteria

The proposed model was evaluated on the NSL-KDD and UNSW-NB15 datasets.

The NSL-KDD dataset was obtained by Tavallaee et al. in 2009 by eliminating duplicate instances in the KDD99 dataset and enabling a more objective reflection of the detection accuracy of the model [33]. It includes DoS, Probe, R2L, and U2R attack types, and has 41 attributes, but the data are extremely unbalanced. It has far fewer attack instances than normal instances, with only 995 R2L attacks and 52 U2R attacks.

The UNSW-NB15 dataset was created by the Cyber Range Lab of the Australian Cyber Security Centre, and includes attack types other than NSL-KDD, i.e., Fuzzers, Analysis, Backdoor, DoS, Exploits, Generic, Reconnaissance, Shellcode, and Worms. Similarly, there are far fewer attack instances than normal instances.

**Figure 5:**
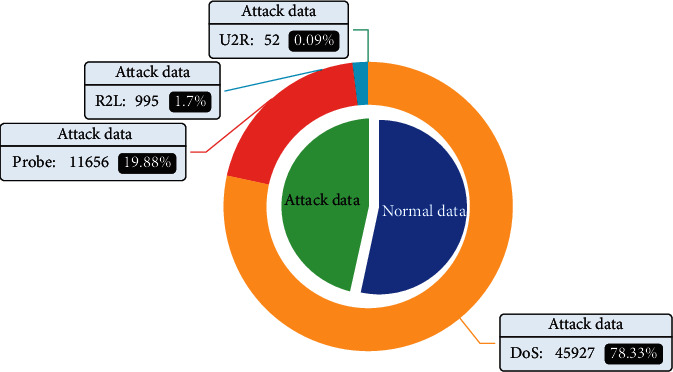
Distribution of NSL-KDD training set types.

The distributions of training set types for the NSL-KDD datasets are shown in Figure 5.

**Figure 6:**
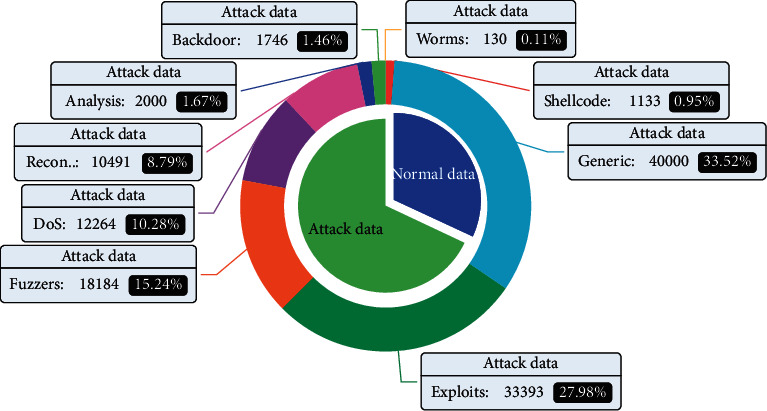
Distribution of UNSW-NB15 training set types.

The distributions of training set types for the UNSW-NB15 datasets are shown in Figure 6.

Comparative experiments used classification accuracy, precision, recall, and F1-score to judge the classification effectiveness of the models. The classification confusion matrix is shown in Table 3.

The four evaluation criteria are as follows:

(14) $$\begin{array}{c} \text{accuracy}=\frac{\text{TP}+\text{TN}}{\text{TP}+\text{TN}+\text{FP}+\text{FN}},\\ \text{recall}=\frac{\text{TP}}{\text{TP}+\text{FN}},\\ \text{precision}=\frac{\text{TP}}{\text{TP}+\text{FP}},\\ F1-\text{score}=2\times \frac{\text{precision}\times \text{recall}}{\text{precision}+\text{recall}}. \end{array}$$

### 4.3. Experimental Results and Analysis

#### 4.3.1. Model Performance Analysis Experiment

To verify the effectiveness of the proposed model at network intrusion detection, we set up performance analysis experiments on network intrusion detection methods based on FCWGAN and BiLSTM.

**Figure 7:**
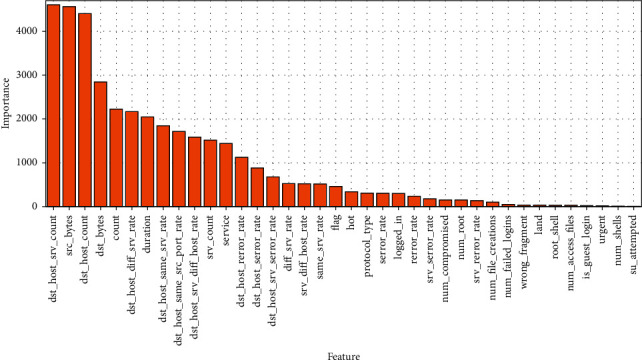
Feature importance map of NSL-KDD.

**Figure 8:**
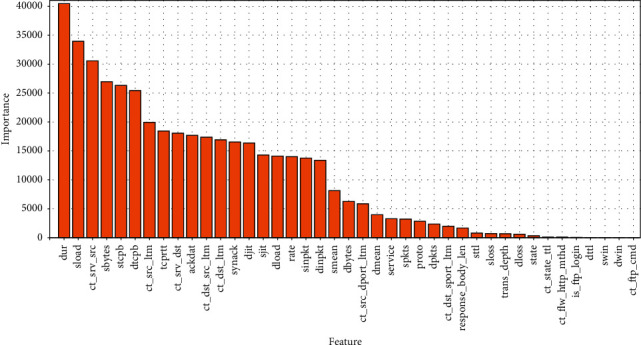
Feature importance map of UNSW-NB15.

FCWGAN was used to select the features of the training set samples of the NSL-KDD and UNSW-NB15 datasets, filter out redundant and useless samples, and simplify the data structure. The feature importance was judged using XGBoost, and the feature importance scores were obtained as shown in Figures 7 and 8.

The feature importance score in Figures 7 and 8 selects the total splitting gain, which can better reflect the importance of variables to the model.

From Figure 7, one can see that among the features of NSL-KDD datasets, the “dst_host_srv_count” is the most important and the “su_attempted” is the lowest; Similarly, it can be seen from Figure 8 that among the features of UNSW-NB15 datasets, the “dur” is the most important and the “ct_ftp_cmd” is the lowest. At the same time, it can be seen that in the above two datasets, the importance of different features varies greatly, and the importance of individual features is close to 0, which has little influence on the discrimination of sample types. Therefore, these useless features with low importance can be screened out to simplify the feature structure.

**Figure 9:**
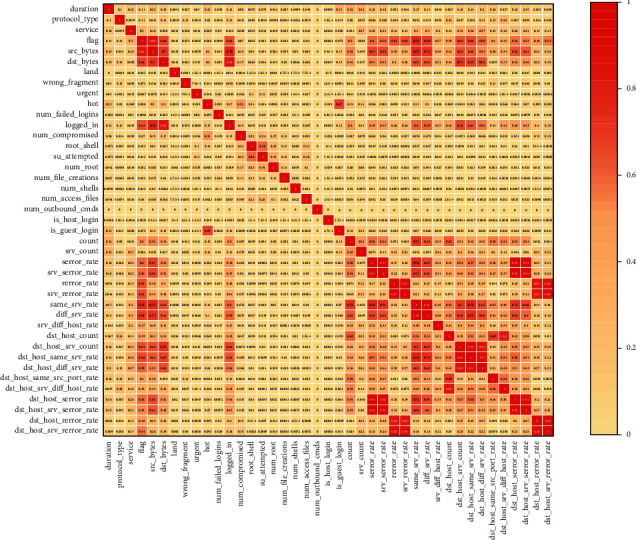
Feature correlation diagram of NSL-KDD.

**Figure 10:**
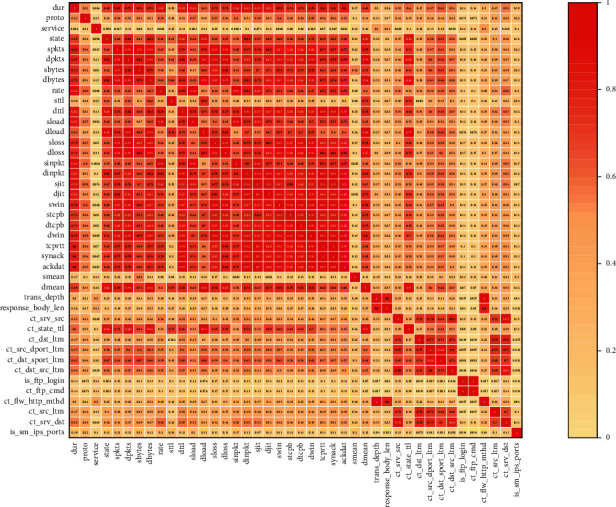
Feature correlation diagram of UNSW-NB15.

The feature correlations were analyzed using the Spearman correlation coefficient; the correlation between individual features is strong, and redundant features can be filtered out (Figures 9 and 10).

We combined the feature importance and correlation for analysis, and the filtered features are shown in Table 4.

Training set samples were then generated based on the selected features. We expanded the training set samples and combined the generated and original samples. The data distribution of the combined training set is shown in Tables 5 and 6.

**Figure 11:**
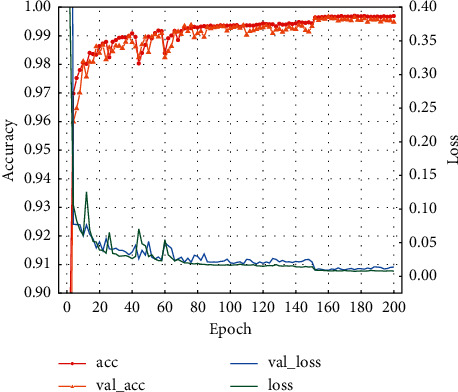
NSL-KDD accuracy curve.

**Figure 12:**
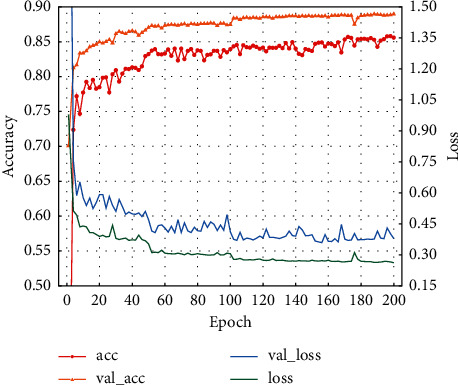
UNSW-NB15 accuracy curve.

Finally, the training set was passed into the BiLSTM network for training, and the test data were passed into the completed model to evaluate the model detection effect. The trends of model detection accuracy and average loss with the number of iterations are shown in Figures 11 and 12.

**Figure 13:**
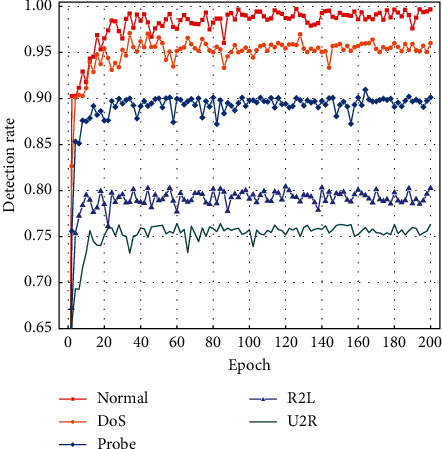
NSL-KDD class detection rate curve.

**Figure 14:**
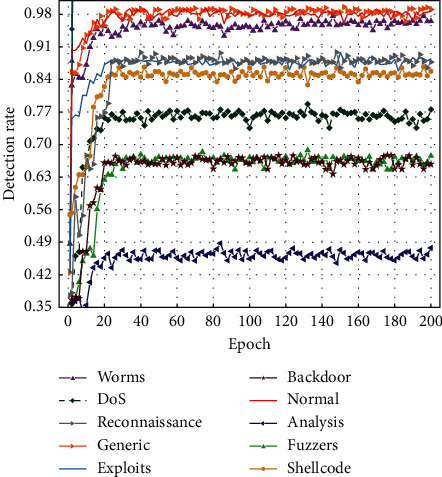
UNSW-NB15 class detection rate curve.

The trends of various class detection rates with the number of iterations are shown in Figures 13 and 14.

From Figures 11 and 12, one can see that the accuracy of the model increases rapidly with the number of iterations at the early stage of training, and gradually stabilizes; the average loss decreases rapidly with the number of iterations, and can reach a stable state quickly. Using the proposed model to perform multiclassification on the NSL-KDD and UNSW-NB15 datasets, the best accuracy rates are 99.57% and 85.59%, respectively. This shows that the model can distinguish types of network intrusion attacks well, thus obtaining high detection accuracy and a better detection effect.

From Figures 13 and 14, it can be seen that the proposed model can accurately identify normal and majority class attacks on both datasets, and the detection rate for minority class attacks can also reach a high standard, showing that the minority class samples generated by the model largely alleviate the impact of the class imbalance problem, thus improving the overall detection effect.

#### 4.3.2. Model Noise Robustness Experiment

In recent years, the network environment has become more and more complex. In addition to a large number of redundant and useless data, there are also noise data in the network data, which will lead to the low robustness of the intrusion detection system [34]. In order to verify the robustness of the model proposed in this paper to noise, this section sets up a noise robustness experiment for network intrusion detection methods based on FCWGAN and BiLSTM.

Different levels of Gaussian white noise are added to NSL-KDD and UNSW-NB15 datasets, which obey *N* (0, 0.02), *N* (0, 0.04), *N* (0, 0.06) and *N* (0, 0.08), respectively. The detection accuracy of the model under the influence of different noise levels is shown in Table 7.

From Table 7, it shows that the accuracy of the two datasets decreases to a certain extent with the increase of the noise level. However, the range of change did not exceed 1.5%. This shows that the model proposed in this paper has strong robustness and stability to the interference of noise, and a small amount of noise data cannot have a significant impact on the performance of the model. At the same time, according to the conclusion of 3.3.1, different levels of Gaussian white noise are added to several features with strong correlation, middle correlation and weak correlation, which tests the accuracy of model detection. The result shows that adding noise to the features with stronger correlation has more obvious impact on the performance of the model, while the features with weaker correlation have little impact. It shows that when dealing with noise, it is not necessary to deal with all features, but only some noise sensitive features, which also confirms the necessity of feature selection.

#### 4.3.3. Model Ablation Experiment

We set up model ablation experiments to verify the proposed feature selection and the ability of CWGAN to improve the detection effect of the model for minority samples.

Under the same experimental conditions, BiLSTM, GAN-BiLSTM, CWGAN-BiLSTM, and the model in this paper were compared on the NSL-KDD dataset. The detection rates of each model for various types of NSL-KDD datasets were evaluated, and are displayed in Table 8.

From Table 8, it shows that the feature extraction and the proposed CWGAN played a relatively significant role in the improvement of the detection rate for minority class samples. The reason is that real-world data contain many irrelevant, redundant, and noisy features, whose removal through feature selection can greatly reduce storage and computational costs, and can simplify the data structure and improve the detection results. The proposed feature selection method was used to directly select a subset of relevant features for the model, eliminate useless and redundant features, and improve the test effectiveness from the original dataset level. CWGAN achieved the controlled generation of minority samples by adding category information and the Wasserstein distance, while avoiding the vanishing gradient, and combining it with the original training set to increase the number of minority samples. Ultimately, this enhanced the training effect of the model. Therefore, we combined the two to process the dataset and improve the test effect of the model.

#### 4.3.4. Comparative Experiments with Different Data Enhancement Algorithms

We set up comparison experiments to verify the superiority of the FCWGAN data enhancement algorithm at network intrusion detection.

Under the same experimental conditions, ROS, ADASYN, SMOTE, WGAN, and the proposed FCWGAN method were used for data enhancement on the NSL-KDD and UNSW-NB15 datasets, respectively, using BiLSTM as a classifier, with test results as shown in Tables 9 and 10.

From Tables 9 and 10, it can be seen that the proposed FCWGAN-BiLSTM achieved the best test results in terms of accuracy, precision, recall, and F1-score. Overall, FCWGAN was better for data enhancement. The time in the table is the training time of a single epoch. It can be found that the training time of the model in this paper is lower than that of other methods, indicating that the calculation speed of the model is the fastest and the calculation cost is the smallest. This is because ROS only performs a simple resampling of the original data, ADASYN and SMOTE perform a random synthesis of the original data based on the k-nearest neighbor principle, and neither learns the nature of the original data. In contrast, FCWGAN, which is based on deep learning, can acquire the potential distribution of the original data, randomly connect the data points with class labels, and pass them to the generator to generate new minority samples. Compared with WGAN, FCWGAN adds feature selection and simplifies the data structure, which calculation cost is reduced and the calculation speed is accelerated. At the same time, a gradient penalty term solves the vanishing gradient problem during training, so that FCWGAN can generate minority class samples that have higher quality and are more similar to the original samples.

#### 4.3.5. Comparative Experiments with Different Classification Algorithms

We performed comparison experiments to verify that BiLSTM could achieve better results for the classification of network intrusions.

Under the same experimental conditions, the dataset was processed using FCWGAN, and was then trained on RF, DNN, LSTM, and BiLSTM. The results of different algorithms for network intrusion behavior were evaluated, and the results are shown in Tables 11 and 12.

From Tables 11 and 12, it can be seen that the proposed FCWGAN-BiLSTM achieved the best results in terms of accuracy, precision, recall, and *F*1-score. Moreover, BiLSTM has advantages in network intrusion detection problem. The reason is that network traffic data have obvious time-series characteristics, while LSTM and BiLSTM have strong time-series processing capability and could perform deeper feature extraction on long-term time-series data. Therefore, this type of method can achieve good results at network intrusion detection. LSTM could only read sequence data from one direction and could not rule out the influence of subsequent information on the detection results. Thus, BiLSTM is used to process the incoming data to improve the detection effect. However, the training time of a single epoch of the model is higher than that of other detection methods, because BiLSTM is more complex than other classification algorithms.

#### 4.3.6. Comparative Experiment with Existing Intrusion Detection Models

Performance comparison experiments were conducted to further verify the comprehensive performance of network intrusion detection algorithms based on FCWGAN and BiLSTM.

Under the same experimental conditions, models with superior detection results in the literature, such as CNN-BiLSTM [35], SSAE-LSTM [36], CWGAN-DNN, and AE-CGAN-RF, were applied to the NSL-KDD and UNSW-NB15 datasets in accordance with their published descriptions and parameter settings, with results as shown in Tables 13 and 14.

From Tables 13 and 14, it can be seen that the proposed model achieved the best detection results on all metrics. Compared with CNN-BiLSTM and SSAE-LSTM, the proposed model uses FCWGAN to simplify the data features and reduce dataset dimensionality, which reduces the computational cost, while generating minority class samples to supplement the dataset, which alleviates the impact of class imbalance, and thus it could obtain better detection results. Compared with CWGAN-DNN and AE-CGAN-RF, the proposed model eliminates high-dimensional disasters and simplifies the data structure, while uses BiLSTM for feature extraction and classification, which can extract more in-depth and comprehensive data features from the time-series level, and thus obtains better multiclassification results.

## 5. Conclusion

To alleviate the impact of class imbalance on the accuracy of network intrusion detection models and improve their effectiveness at detecting network intrusion attacks, we proposed a network intrusion method based on FCWGAN and BiLSTM. The method uses XGBoost and Spearman correlation coefficients to process the dataset, which effectively filters out redundant and useless features, simplifies the data structure, which reduces the computational cost and training time, and avoids high-dimensional disasters. Minority class samples are generated using CWGANs to supplement the dataset and alleviate class imbalance. BiLSTM is used to extract the time-series features of data to complete the classification of network intrusions. Extensive experiments on the NSL-KDD and UNSW-NB15 datasets demonstrated that the model greatly improves the detection effect for minority class samples, has a strong feature extraction capability, high detection accuracy, and low false-positive rate when processing large-scale network data, and shows promise for real-time intrusion detection systems. However, the accuracy of this model on the UNSW-NB15 dataset demonstrated that there is room for improvement. Future work will focus on this deficiency, and we will investigate the construction of feature extraction and classification models to find ways to improve detection accuracy.

## Figures and Tables

**Table 1:** CWGAN training algorithm.

Algorithm 1: minority class sample generation based on CWGANs
Input: *s*=(*z*, *y*), where *z* is noise data, *y* is class label
Output: *s*_*G*_=[*G*(*z*, *y*′), *y*′]
(1) **While** *D* **does** **not** **approach** 0.5 /^∗^CWGAN training^∗^/
(2) **for** *t* =1,…, *n* **do** /^∗^optimize discriminator^∗^/
(3) Sampling {(*x*_*i*_, *y*_*i*_)}_*i*=1_^*n*_*z*_^ from *p*_data_(*x*, *y*)
(4) Sampling {(*z*_*i*_)}_*i*=1_^*n*_*z*_^ from *p*_*z*_(*z*)
(5) $\eta_{\theta_{D}}\leftarrow \nabla \theta_{D}\left[1/n_{z}\sum_{i=1}^{n_{z}}\left\{\begin{array}{c} D\left(x_{i},y_{i}\right)-D\left(G\left(z_{i},y_{i}^{\prime }\right),y_{i}^{\prime }\right)-\\ \lambda E_{\left(x,y\right)∼p_{\text{Penaty}}}{\left[\left\|\nabla_{\left(x,y\right)}D\left(x,y\right)\right\|-1\right]}^{2} \end{array}\right\}\right]$
(6) *θ*_*D*_ ← *θ*_*D*_+*α*_*D*_*·*Adam(*θ*_*D*_, *η*_*θ*_*D*__)
(7) **end**
(8) from *p*_*z*_(*z*) sample {(*z*_*i*_)}_*i*=1_^*n*_*z*_^ /^∗^optimize generator^∗^/
(9) *η*_*θ*_*G*__ ← ∇*θ*_*G*_[1/*n*_*z*_∑_*i*=1_^*n*_*z*_^(*D*(*G*(*z*_*i*_, *y*_*i*_′), *y*_*i*_′))]
(10) *θ*_*G*_ ← *θ*_*G*_ − *α*_*G*_*·*Adam(*θ*_*G*_, *η*_*θ*_*G*__)
(11) **end**
(12) **return** *u* /^∗^generate samples^∗^/

where *θ*_*G*_, *η*_*θ*_*G*__, *θ*_*D*_ and  *η*_*θ*_*D*__ respectively denote the network parameters and gradients of the generator and discriminator.

**Table 2:** Model parameter settings.

Parameter	Setting
XGBoost maximum depth	12
XGBoost gamma value	0
CWGAN learning rate	0.0001
CWGAN training iterations	200
Noise dimension	32
Batch size setting	1024
Loss function	Categorical cross-entropy
Optimizer	Nadam
Optimizer learning rate	0.001
BiLSTM cell count	64/128
Dropout rate	0.5

**Table 3:** Definition of classification confusion matrix.

		Predicted class	
		Normal	Abnormal
Actual class	Normal	TP	FN
	Abnormal	FP	TN

**Table 4:** Feature selection results.

Dataset	Feature selection	Number
NSL-KDD	duration, protocol_type, service, dst_host_srv_count, src_bytes, dst_host_count, dst_bytes, count, dst_host_same_src_port_rate, dst_host_srv_diff_host_rate, srv_count, dst_host_rerror_rate, dst_host_serror_rate, diff_srv_rate, srv_diff_host_rate, hot, serror_rate, rerror_rate, num_compromised, num_root	20

UNSW-NB15	dur, sload, ct_srv_src, sbytes, stcpd, ct_src_ltm, tcprtt, ct_srv_dst, ct_dst_src_ltm, ct_dst_ltm, djit, sjit, dload, smean, ct_src_dport_ltm, dmean, service, proto, response_body_len	19

**Table 5:** Distribution of NSL-KDD dataset before and after sample generation.

Class	Before sample generation	After sample generation
Normal	67343	67343
DoS	45927	45927
Probe	11656	11656
R2L	995	5995
U2R	52	5052

**Table 6:** Distribution of UNSW-NB15 dataset before and after sample generation.

Class	Before sample generation	After sample generation
Normal	56000	56000
Generic	40000	40000
Exploits	33393	33393
Fuzzers	18184	18184
DoS	12264	12264
Reconnaissance	10491	10491
Analysis	2000	7000
Backdoor	1746	6746
Shellcode	1133	6133
Worms	130	5130

**Table 7:** Detection accuracy under the influence of different noise levels.

Dataset	Noise level				
	0	0.02	0.04	0.06	0.08
NSL-KDD	99.57 ± 0.21	99.55 ± 0.22	99.45 ± 0.22	98.88 ± 0.24	98.27 ± 0.25
UNSW-NB15	85.59 ± 0.27	85.53 ± 0.29	85.28 ± 0.30	84.71 ± 0.31	84.15 ± 0.33

**Table 8:** Ablation experiment detection rate of various types.

Algorithm	Type of samples				
	Normal	DoS	Probe	U2R	R2L
BiLSTM	94.65 ± 0.21	88.24 ± 0.19	72.91 ± 0.23	46.81 ± 0.35	51.97 ± 0.30
GAN-BiLSTM	95.31 ± 0.25	92.18 ± 0.22	81.27 ± 0.30	60.33 ± 0.42	65.10 ± 0.37
CWGAN-BiLSTM	98.54 ± 0.19	94.60 ± 0.15	85.15 ± 0.23	70.20 ± 0.26	72.13 ± 0.25
Model in this paper	99.68 ± 0.14	96.01 ± 0.11	90.12 ± 0.15	76.35 ± 0.27	80.26 ± 0.19

**Table 9:** Comparison of data enhancement algorithms on NSL-KDD dataset.

Algorithm	Evaluation metrics				
	Accuracy	Precision	Recall	*F*1-score	Time (s)
ROS-BiLSTM	89.18 ± 0.35	90.34 ± 0.40	88.61 ± 0.35	89.46 ± 0.37	4
ADASYN-BiLSTM	92.95 ± 0.24	93.12 ± 0.27	92.61 ± 0.21	92.86 ± 0.25	5
SMOTE-BiLSTM	93.66 ± 0.28	94.63 ± 0.34	93.14 ± 0.26	93.88 ± 0.30	3
WGAN-BiLSTM	96.56 ± 0.23	96.71 ± 0.28	95.65 ± 0.21	96.20 ± 0.26	7
Model in this paper	99.57 ± 0.21	99.55 ± 0.20	99.47 ± 0.17	99.51 ± 0.18	2

**Table 10:** Comparison of data enhancement algorithms on UNSW-NB15 dataset.

Algorithm	Evaluation metrics				
	Accuracy	Precision	Recall	*F*1-score	Time (s)
ROS-BiLSTM	81.70 ± 0.43	79.32 ± 0.47	80.49 ± 0.41	79.90 ± 0.44	6
ADASYN-BiLSTM	83.65 ± 0.37	84.11 ± 0.40	82.14 ± 0.35	83.12 ± 0.37	6
SMOTE-BiLSTM	83.66 ± 0.31	84.28 ± 0.34	81.24 ± 0.27	82.73 ± 0.30	5
WGAN-BiLSTM	81.49 ± 0.30	84.71 ± 0.24	82.51 ± 0.28	83.60 ± 0.26	8
Model in this paper	85.59 ± 0.27	86.11 ± 0.21	85.57 ± 0.24	85.84 ± 0.22	4

**Table 11:** Comparison of classification algorithms on NSL-KDD dataset.

Algorithm	Evaluation metrics				
	Accuracy	Precision	Recall	*F*1-score	Time (s)
FCWGAN-RF	91.29 ± 0.27	90.24 ± 0.29	89.11 ± 0.21	89.67 ± 0.24	1
FCWGAN-DNN	95.11 ± 0.23	96.01 ± 0.22	94.98 ± 0.17	95.00 ± 0.19	2
FCWGAN-LSTM	98.29 ± 0.23	98.37 ± 0.21	98.14 ± 0.15	98.25 ± 0.18	2
Model in this paper	99.57 ± 0.21	99.55 ± 0.20	99.47 ± 0.17	99.51 ± 0.18	2

**Table 12:** Comparison of classification algorithms on UNSW-NB15 dataset.

Algorithm	Evaluation metrics				
	Accuracy	Precision	Recall	*F*1-score	Time (s)
FCWGAN-RF	81.00 ± 0.37	81.94 ± 0.33	80.97 ± 0.31	81.45 ± 0.32	1
FCWGAN-DNN	83.44 ± 0.31	84.12 ± 0.33	83.40 ± 0.27	83.76 ± 0.30	2
FCWGAN-LSTM	84.98 ± 0.30	85.44 ± 0.29	84.67 ± 0.25	85.05 ± 0.28	3
Model in this paper	85.59 ± 0.27	86.11 ± 0.21	85.57 ± 0.24	85.84 ± 0.22	4

**Table 13:** Comparison of multiclassification on NSL-KDD dataset.

Algorithm	Evaluation metrics				
	Accuracy	Precision	Recall	*F*1-score	Time (s)
CNN-BiLSTM	99.22 ± 0.31	99.18 ± 0.29	99.14 ± 0.24	99.15 ± 0.26	6
SSAE-LSTM	97.63 ± 0.34	97.91 ± 0.33	97.21 ± 0.28	97.56 ± 0.30	4
CWGAN-DNN	98.13 ± 0.26	99.03 ± 0.30	97.91 ± 0.25	98.46 ± 0.27	8
AE-CGAN-RF	98.53 ± 0.27	98.67 ± 0.28	98.31 ± 0.23	98.49 ± 0.25	7
Model in this paper	99.57 ± 0.21	99.55 ± 0.20	99.47 ± 0.17	99.51 ± 0.18	2

**Table 14:** Comparison of multiclassification on UNSW-NB15 dataset.

Algorithm	Evaluation metrics				
	Accuracy	Precision	Recall	*F*1-score	Time (s)
CNN-BiLSTM	82.08 ± 0.43	82.68 ± 0.43	80.00 ± 0.37	81.32 ± 0.40	10
SSAE-LSTM	82.31 ± 0.45	83.65 ± 0.44	81.94 ± 0.36	82.78 ± 0.41	7
CWGAN-DNN	82.61 ± 0.37	82.95 ± 0.41	82.11 ± 0.33	82.53 ± 0.38	14
AE-CGAN-RF	81.24 ± 0.39	83.47 ± 0.40	80.31 ± 0.35	81.86 ± 0.38	13
Model in this paper	85.59 ± 0.27	86.11 ± 0.21	85.57 ± 0.24	85.84 ± 0.22	4

## Data Availability

All data used in this paper can be obtained by contacting the authors of this study.
